# Immunomodulatory Effects of *Escherichia coli* Phage GADS24 on Human Dendritic Cells

**DOI:** 10.3390/biomedicines13071519

**Published:** 2025-06-21

**Authors:** Alia M. Aldahlawi, Ghadah A. Alsubhi, Jehan S. Alrahimi, Fatemah S. Basingab, Kawther A. Zaher

**Affiliations:** 1 Department of Biological Sciences, Faculty of Science, King Abdulaziz University, Jeddah 215589, Saudi Arabia; 2 Immunology Unit, King Fahd Medical Research Center, King Abdulaziz University, Jeddah 21589, Saudi Arabia; 3 Department of Medical Laboratory Sciences, Faculty of Applied Medical Sciences, King Abdulaziz University, Jeddah 21589, Saudi Arabia

**Keywords:** bacteriophage therapy, multidrug-resistant bacteria, dendritic cells, immunomodulation, GADS24, cytokines, flow cytometry

## Abstract

**Background:** Multidrug-resistant (MDR) *Escherichia coli* (*E. coli*) strains pose a significant public health challenge, which has led to the exploration of alternative therapeutic strategies. Due to their antibacterial and immunomodulatory properties, bacteriophages have emerged as promising therapeutic agents. **Methods:** This study investigates the effects of GADS24, a novel lytic bacteriophage of *E. coli*, on human-monocyte-derived dendritic cells (DCs). DCs are exposed to purified GADS24 phage, bacterial lysate, or a combination of both. Flow cytometry was used to assess the expression of surface markers (HLA-DR, CD80, CD83, and CD86), and ELISA was used to measure cytokine production (IL-10 and IL-12p70). **Results:** Following treatment with bacterial lysate, a significant increase in DC maturation markers was observed. The GADS24 phage alone induced a moderate upregulation of these markers, decreased IL-10 secretion, and increased IL-12p70. Combining bacterial lysate and phage tempered the maturation response compared to the lysate treatment alone. **Conclusion:** These findings suggest that GADS24 exerts antibacterial activity and modulates host immunity by influencing DC maturation and cytokine production. Due to its dual antimicrobial and immunomodulatory functions, GADS24 is likely to be a valuable adjunctive therapy for multidrug-resistant (MDR) bacterial infections. Furthermore, in vivo studies are necessary to confirm these promising in vitro results.

## 1. Introduction

Global antimicrobial resistance (AMR) poses a significant threat to public health, prompting researchers to explore alternative treatments such as phage therapy [1]. *E. coli* is one of the most concerning pathogens, since it can lead to a wide range of extraintestinal infections and increased resistance to several antibiotics [2]. As an alternative or supplementary antimicrobial agent in the fight against multidrug-resistant (MDR) bacteria, bacteriophages (phages), which are viruses that specifically infect and lyse bacteria, have recently gained attention [3].

Compared with traditional antibiotics, phage therapy offers several advantages, including specificity for target bacteria, minimal disruption of the host microbiota, and the ability to replicate at the site of infection [4]. Furthermore, phages are self-limiting; as the bacterial host population declines, the phage population decreases, thus potentially reducing collateral damage to the host [5]. Despite its promising results in preclinical models and early clinical trials, the full immunological consequences of phage therapy remain unclear [6].

Significantly, bacteriophages can interact with the mammalian host immune system, either directly or indirectly. Innate immune receptors can directly recognize phages, while indirectly, they can provoke the release of bacterial components, such as lipopolysaccharides (LPSs), through bacterial lysis, thereby influencing immune activation [7]. Such interactions can modulate cytokine secretion, antigen presentation, and inflammatory pathways, which must be carefully considered when designing phage-based therapies [8].

Immune responses are initiated and regulated by dendritic cells (DCs). Through pattern-recognition receptors, DCs can detect microbial pathogens, process antigens, and activate T cells, thus bridging innate and adaptive immunity [8]. Changes in DC function, including changes in surface marker expression and cytokine profiles, can significantly alter the nature and balance of immune responses.

Although phage therapy has significant potential, it is limited by several factors, including immunogenicity, which may affect phage pharmacokinetics, biodistribution, and therapeutic effectiveness [9]. Understanding how phages influence dendritic cell biology is crucial for developing safe and effective phage therapies that harness their antibacterial properties while minimizing unintended immunopathological effects.

ESKAPEE pathogens (*Enterococcus faecium*, *Staphylococcus aureus*, *Klebsiella pneumoniae*, *Acinetobacter baumannii*, *Pseudomonas aeruginosa*, *Enterobacter* spp., and *Escherichia coli*) represent the leading causes of multidrug-resistant infections globally, particularly in nosocomial settings. These bacteria are notorious for their capacity to develop and share resistance mechanisms, complicating treatment protocols [10]. Bacteriophage-based strategies have been increasingly explored as a countermeasure to these threats. For instance, lytic phages against *K. pneumoniae* and *A. baumannii* have demonstrated promising results in preclinical models by significantly reducing bacterial load and inflammation [11]. Similarly, *P. aeruginosa* phages have been evaluated in cystic fibrosis and wound infections with favorable safety and efficacy outcomes [12]. Phages targeting *Escherichia coli* (*E. coli*), including those from the *Myoviridae* and *Siphoviridae* families, have been extensively studied for urinary tract infections and gastrointestinal diseases [13]. Despite progress, more studies are needed to explore their immunological interactions. Our analysis focuses on a novel *E. coli* phage, GADS24, and its immunomodulatory effects on human dendritic cells, thereby contributing to the broader effort of understanding phage–host immune interactions across ESKAPEE pathogens.

Despite the expanding interest in bacteriophages as alternatives to antibiotics, most studies focus on their lytic activity, overlooking their potential immunomodulatory effects. The GADS24 phage used in this study is a novel isolate targeting *E. coli*, and its immunological impact has not been previously reported. Unlike conventional phage characterizations, this work investigates not only the antimicrobial efficacy of GADS24 but also its influence on DC maturation and cytokine secretion. This dual focus enables a more comprehensive understanding of phage–host interactions, particularly as immune modulation becomes a crucial consideration in phage therapy design. This study evaluates the effects of GADS24, a novel *E. coli* phage whose genome has been publicly deposited in GenBank (accession number: OQ703618). Based on the emerging evidence that bacteriophages may interact with components of the host immune system, we hypothesized that the newly isolated GADS24 phage would not only exhibit bacteriolytic activity but also modulate dendritic cell phenotype and cytokine secretion. This study aimed to evaluate the extent of these immunomodulatory effects using primary human-monocyte-derived dendritic cells as a model. By conducting this study, we hope to contribute to a better understanding of the immunological consequences of phage therapy.

## 2. Materials and Methods

### 2.1. Bacterial Strains and Phage

An *E. coli* bacteriophage, GADS24, was obtained from the Immunology Unit of King Fahd Medical Research Centre, King Abdulaziz University, Saudi Arabia. GADS24’s genome sequence was submitted to the National Center for Biotechnology Information GenBank database, and the accession number OQ703618 (txid3038244) was assigned [14].

According to an established protocol [15], the phage was purified using polyethylene glycol (PEG) 8000. A total of 30 mL of phage stock was combined with 7.5 mL of 20% PEG 8000 and incubated on ice for 30 min before centrifugation twice at 6000× *g* for 20 min. After resuspending the phage pellet in 1 mL of SM buffer, it was transferred to a microcentrifuge tube, followed by a final centrifugation at 11,000× *g* for 10 min.

The *Escherichia coli* strain NRT114 [16] was used as the primary host for bacteriophage work, as it is the reference host for the novel lytic phage GADS24. The bacterial strain was kindly provided by Sana Khalifa Alsheikh, King Fahad Medical Research Center (KFMRC), King Abdulaziz University, Jeddah, Saudi Arabia.

Phage titration was performed using the standard double-layer plaque assay method as described by Bonilla et al. [17], with modifications to improve clarity and reproducibility. *Escherichia coli* NRT114 was cultured in LB broth overnight at 37 °C with shaking (1000× *g*). A 1:100 dilution of the overnight culture was then prepared and grown to mid-log phase, reaching an optical density of 0.697 at 600 nm (OD_600_). Ten-fold serial dilutions of the phage–bacterial lysate (from 10^−1^ to 10^−10^) were prepared in SM buffer. For each dilution, 100 µL of purified phage suspension was mixed with 200 µL of the mid-log *E. coli* culture and incubated at room temperature for 10 min to allow for phage adsorption. The mixture was then combined with 3 mL of molten soft LB agar (0.7% agar, cooled to ~45 °C) and immediately poured onto solid LB agar plates. The plates were incubated overnight at 37 °C. Plaques were counted at the dilution yielding 30–300 plaques, and the phage titer was calculated as plaque-forming units per milliliter (PFU/mL).

For host range analysis, ten additional *E. coli* strains with varying antibiotic resistance profiles were also used in the host range test. All *Escherichia coli* strains included in this study were provided by the Special Infectious Disease Unit, King Fahd Medical Research Center, King Abdulaziz University, Jeddah, Saudi Arabia. Each strain was identified and confirmed through standard biochemical tests, and its genetic uniqueness was further validated by sequencing the 16S rRNA gene to eliminate duplicates.

### 2.2. Spot Assay

A spot assay was performed to assess the phage’s ability to infect *E. coli*, following Bonilla et al. [17]. Briefly, 300 µL of mid-log phase bacteria (OD_600_ = 0.568) was mixed with 3 mL of 0.6% (*w*/*v*) LB soft agar and overlaid onto solid LB agar plates. Two 10 µL drops of phage suspension were spotted onto the lawn and incubated overnight at 37 °C. Zones of lysis were observed and photographed the following day using a colony counter.

### 2.3. Bacteria and Bacterial Lysate Preparation

The *Escherichia coli* strain NRT114 was used for the preparation of bacterial lysate [17,18]. A single colony was cultured overnight on LB agar at 37 °C. The next day, 20 mL of LB broth was inoculated with the culture and incubated at 37 °C with shaking at 180 rpm for 4 h until the culture reached the mid-log phase (OD_600_~0.6). Then, 300 µL of purified GADS24 phage (10^8^ PFU/mL) was added and incubated at 37 °C for five hours to ensure complete lysis. The lysate was centrifuged at 6000× *g* for 10 min. The supernatant was then filtered through a 0.22 µm membrane filter to remove residual bacteria and, subsequently, heat-treated at 70 °C for 30 min to inactivate any remaining active phage particles. This mild heat treatment was selected to ensure phage inactivation while minimizing denaturation of bacterial components relevant for dendritic cell stimulation. The inactivated lysate was stored at 4 °C until use. Complete inactivation of the phage was confirmed using a spot assay, as described previously [17].

For co-treatment experiments, fresh purified phage GADS24 (10^8^ PFU/mL) was added back to the sterile lysate before dendritic cell exposure. This allowed us to distinguish between the immunomodulatory effects of phage alone, bacterial lysate alone, and the synergistic interaction of a calculated dose of the two.

### 2.4. Quantification of LPS Levels

To obtain quantitative LPS levels, the Pierce^TM^ LAL Chromogenic Endotoxin Quantitation Kit (Thermo Scientific™, catalog number: A39552S, Waltham, MA, USA) was used according to the manufacturer’s instructions.

To reduce LPS contamination from the bacteriophage preparation, 1-octanol (Sigma-Aldrich, Merck, St. Louis, MO, USA; cat. no. 820931) was used as previously described [12]. Briefly, equal volumes of phage suspension and 1-octanol were mixed and incubated, and the aqueous phase was then dialyzed overnight at 4 °C to remove residual solvent and LPSs. The organic solvent was removed by the dialysis method using Spectrum™ Spectra/Por™ (Fisher Scientific, Hampton, NH, USA; product code: 11425849) and performed according to the manufacturer’s instructions. The aqueous phase was collected and dialyzed overnight at 4 °C against sterile phosphate-buffered saline (PBS) using a 10 kDa MWCO membrane to remove residual solvent and endotoxins. Phage and bacterial lysates were both prepared by infecting *E. coli* NRT114 with GADS24, followed by purification and inactivation steps as described.

### 2.5. Host Range Test

*Spot assays* were conducted to determine the range of hosts the lytic phage GADS24 can infect. As a result of overnight incubation at 37 °C, the results were classified as either no plaques (−) or clear plaques (+) based on the clarity of the spots. All host range tests were performed in triplicate.

### 2.6. Isolation and Differentiation of Monocytes from Peripheral Blood Mononuclear Cells (PBMCs)

#### 2.6.1. Ethical Approval

The study protocol was evaluated and approved by the Research Ethics Committee of the Unit of Biomedical Ethics, King Abdulaziz University (reference no. 501-21). Donors included healthy male and female volunteers from diverse ethnic backgrounds, all with no recent history of infection, chronic illness, or antibiotic use in the past three months. After signing a written informed consent form, they voluntarily provided blood for the study.

#### 2.6.2. DC Generation and Treatment

Peripheral blood mononuclear cells (PBMCs) were obtained from three healthy adult donors, aged between 25 and 32 years, following the described method [19]. Briefly, cells were plated at a concentration of 1 × 10^6^ cells/mL in six-well plates using a differentiation medium containing IL-4 (Solarbio^®^, Recombinant human interleukin 4; catalog number P00009, Beijing, China) and GM-CSF (Solarbio^®^, Recombinant human granulocyte-macrophage colony-stimulating factor; catalog number: P00130, Beijing, China). As established in prior protocols, DCs were differentiated using 50 ng/mL GM-CSF and 20 ng/mL IL-4 [20]. The medium was replenished with the same concentration of cytokines on day four. On day seven, cells in the wells were treated with different substances, as follows: a negative control (untreated cells) and a positive control (activated using LPSs) (catalog number: L2630-25MG, Sigma-Aldrich^®^, St. Louis, MO, USA). Immature DCs (imDCs) were cultured with 10 µL of LPSs (at a final concentration of 1 μg/mL) for activation, phage treatment (GADS24 phage at 1 × 10^8^ PFU/mL, and the residual LPS level was measured at 0.04 EU/mL), bacterial lysate treatment (10 µL), and a combination of phages and bacterial lysate (10 µL each, and the residual LPS level was measured at 0.37 EU/mL). This volume is a well-established standard in phage research and contributes to the robustness of the experimental design [20]. Cell morphology was examined using an inverted microscope (Nikon Eclipse Ti-S, Tokyo, Japan) with digital camera software (Nikon’s Digital Sight DS-U3).

### 2.7. Flow Cytometry Analysis

At 24 h post-treatment, cells were stained with fluorochrome-conjugated antibodies targeting CD14 (Solarbio^®^, antihuman CD14-FITC; catalog number: K010165M, Beijing, China), CD80 (Solarbio^®^, antihuman CD80-FITC; catalog number: K010166M, Beijing, China), CD83 (Solarbio^®^, antihuman CD83-FITC; catalog number: K010167M, Beijing, China), CD86 (Solarbio^®^, antihuman CD86-FITC; catalog number: K010164M, Beijing, China), and HLA-DR (Solarbio^®^, antihuman HLA-DRA-FITC; catalog number: K010170M, Beijing, China). To assess the potential cytotoxicity of the phage or lysate exposure, we monitored cell viability using propidium iodide (PI) exclusion during flow cytometry. Across all treatment groups, ≥91% of cells remained PI-negative (viable), indicating that neither the phage GADS24 nor the bacterial lysate induced significant cell death. These results support that the observed immunophenotypic changes were not due to cytotoxic effects. Live cells were gated based on FSC/SSC morphology and PI exclusion. Marker-positive populations were quantified using unstained and propidium iodide (PI) staining controls to define gating thresholds, which were used to exclude dead cells during analysis. Surface marker expression was assessed, and representative flow cytometry plots are shown in Supplementary Figure S1. The results are based on analyzing at least 10,000 cells, and the percentage of positively labeled cells is calculated. Data were acquired using a flow cytometer (Beckman Coulter Life Sciences, Indianapolis, IN, USA) and analyzed with FCS Express 7 software (De Novo Software, Ontario, CA, USA).

### 2.8. Cytokines Assessment

Cell culture supernatants were collected to measure IL-10 and IL-12 cytokine levels using ELISA. The ELISA kits for IL-10 (Solarbio^®^; cat. no. SEKH-0018, Beijing, China) and IL-12 (p70) (Solarbio^®^; cat. no. SEKH-0021, Beijing, China) were purchased from Solarbio^®^. The procedure was performed according to the manufacturer’s instructions. Cell culture supernatants were harvested after 24 h of stimulation and stored at –80 °C until cytokine analysis.

### 2.9. Statistical Analysis

Data were analyzed using one-way ANOVA with a significance level (α) of 0.05. A *t*-test was used to compare the treatment groups with the negative control, and all selected participants were healthy. A post hoc Tukey HSD test was chosen to identify significant differences among multiple treatment groups while controlling for Type I errors. This test is appropriate given the study’s design, which involves pairwise comparisons under the assumption of normality and homogeneity of variance. The post hoc comparisons were selected based on the study’s primary objectives, which focused on evaluating the effects of bacterial lysate, phages, and their combination on DC marker expression. Specifically, comparisons were made between the control and treatment groups.

All experiments were performed in triplicate (n = 3) unless otherwise stated. Data are expressed as the mean ± standard deviation (SD). Statistical comparisons across multiple treatment groups were performed using one-way analysis of variance (ANOVA) followed by Tukey’s honestly significant difference (HSD) post hoc test to determine pairwise group differences.

A *p*-value of less than 0.05 was considered statistically significant. GraphPad Prism (version 9.0, GraphPad Software, San Diego, CA, USA) was used for all statistical analyses and data visualization.

## 3. Results

### 3.1. Host Range

**Figure 1:**
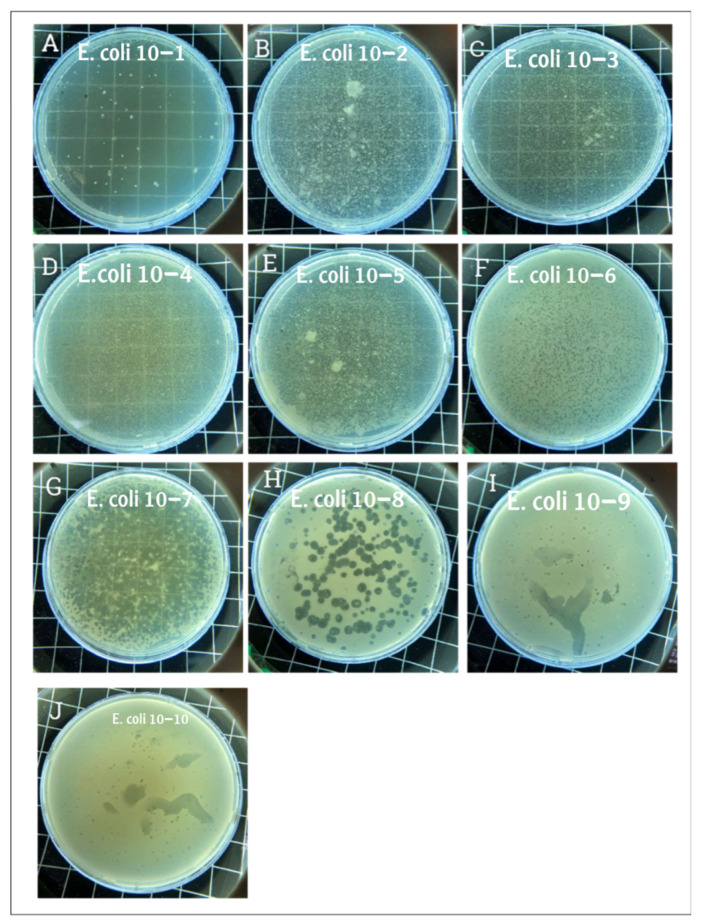
Plaque assay results. Panel (**A**) displays the plaque morphology where the phage dilution was the lowest, and the phage completely lysed the *E. coli*. Panels (**B**–**E**) show a slightly transparent plate where the phage dilution was higher. Panels (**F**,**G**) show hundreds of plaques corresponding to phage dilutions of 10^−6^ and 10^−7^, respectively. Panel (**H**) shows clear, countable, and rounded plaques. Panels (**I**,**J**) show small dots where the phage concentrations were 10^−9^ and 10^−10^, respectively. The phage solution was serially diluted from 10^−1^ to 10^−10^, and plaques were observed even at the 10^−9^ and 10^−10^ dilutions, indicating high infectivity and lytic potential of GADS24.

Host range testing of phage GADS24 was carried out using the spot test method against a panel of multidrug-resistant *E. coli* strains. Each test was performed in triplicate using independent phage and bacterial preparations to confirm consistency. Plates were incubated overnight at 37 °C, and lytic activity was recorded as clear or turbid lysis zones (Figure 1). Five out of ten strains exhibited lytic activity upon phage infection (Table 1) ((+) indicates apparent lysis, and (−) indicates no lysis).

### 3.2. Phage Titration

The plaque Assay aimed to assess the phage’s capacity to lyse *E. coli* and determine its titer. Upon examination, large, clear plaques were observed at high phage concentrations (low dilution), while small dots of plaques were observed at lower concentrations (higher dilution), confirming phage-mediated lysis (Figure 1). Clear circular plaques were counted manually, and titers were calculated as PFU/mL. The figure description clearly explains what each panel (Figure 1A–J) represents, showing serial dilutions and lysis. This figure appears to support titration and lytic activity.

### 3.3. Morphology of Human-Monocyte-Derived Dendritic Cells

**Figure 2:**
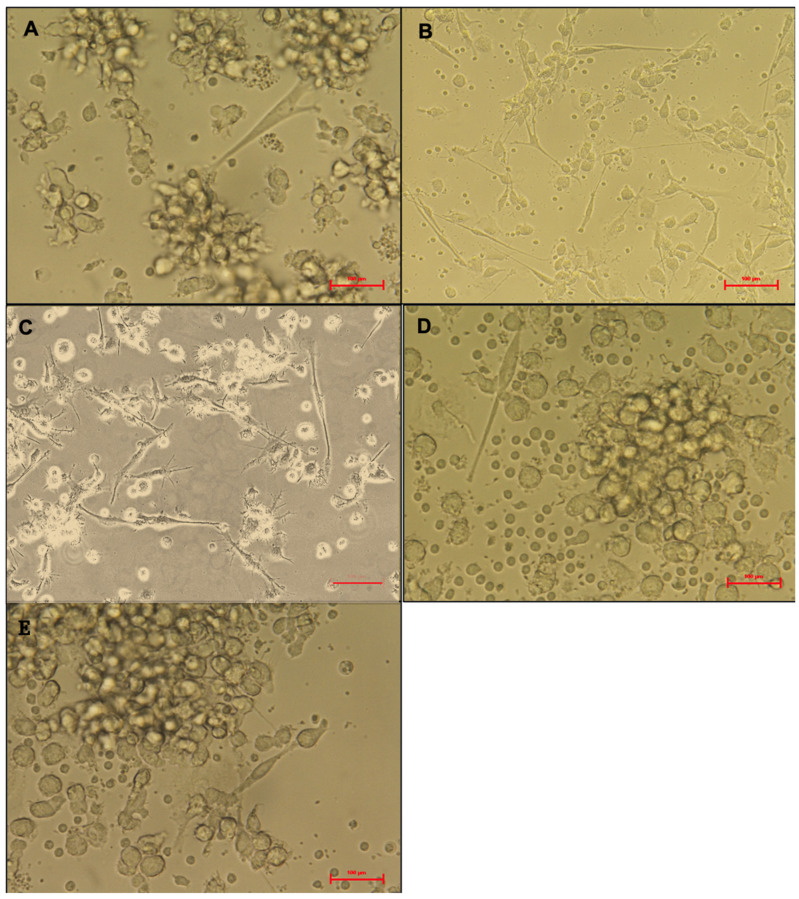
Morphology of treated dendritic cells (DCs). Untreated DCs (negative control) (**A**), LPS-treated DCs (**B**), bacterial-lysate-treated DCs (**C**), phage-treated DCs (**D**), phage with bacterial-lysate-treated DCs (**E**). the imDCs display a semi-rounded, elongated, and irregular form with a short cytoplasmic extension. LPS-stimulated imDCs show an irregular shape with extended cytoplasmic extensions and increased dendrites. Images are captured using an inverted microscope at 40× magnification. Each treatment was performed in triplicate using cells from three independent donors.

An inverted microscope (Nikon Eclipse Ti Inverted Microscope, Nikon Instruments Inc., Tokyo, Japan) with digital camera software was used to monitor monocyte morphological changes during the culture period. The distinction between mature and immature DCs was evident by day eight (Figure 2). Immature DCs appeared more rounded with shorter cytoplasmic projections (Figure 2A), whereas mature DCs exhibited irregular, elongated forms with longer cytoplasmic projections (Figure 2B). Compared with the LPS-treated DCs, the phage- and bacterial-lysate-treated DCs displayed a minor morphological change (Figure 2C–E). No significant reduction was observed in the number of cells with long dendrites, maintaining approximately 0.8 × 10^6^ per well. Few cells possessed elongated forms, and most cells appeared as rounded clusters. The images aim to illustrate morphological differences between immature DCs and mature DCs under the four different treatments (Figure 2A–E).

### 3.4. Surface Marker Expression

On day eight, cells were collected, labeled with monoclonal antibodies, and assessed using flow cytometry. An unstained control was used to adjust for the auto-fluorescence background and adequately configure the voltages and negative gates as a secondary negative control. The flow cytometry analysis demonstrated effective surface marker profiling. Dead cells were excluded based on propidium iodide (PI) staining. The gating strategy used for the flow cytometric analysis is illustrated in Supplementary Figure S1. Briefly, debris and dead cells were excluded based on the forward/side scatter and PI staining. HLA-DR^+^ events were gated as mature dendritic cells for quantification of surface marker expression.

The positive surface marker expression was subsequently analyzed. CD14+-identified dendritic cells, as well as the expression of HLA-DR, CD80, CD83, and CD86, were quantified. Gating thresholds were established based on isotype controls and unstained cells. Surface indicators of mature DCs, including HLA-DR (a major histocompatibility complex (MHC) class II molecule), CD80, CD86, and CD83, were significantly expressed, while CD14 (a monocyte marker) exhibited low or no expression. Each treatment was performed using cells from three independent donors in triplicate. Cells were initially gated based on forward-scatter (FSC) and side-scatter (SSC) characteristics to exclude cellular debris. Viable cells were subsequently selected by gating on PI-negative populations, thereby excluding dead cells. From the viable cell gate, dendritic cells were identified by their reduced CD14 expression profile. Fluorescence gates for dendritic cell surface markers (HLA-DR, CD80, CD83, and CD86) were defined using unstained and isotype controls, clearly distinguishing positive populations from negative populations. A representative gating strategy illustrating these sequential gating steps is provided in Supplementary Figure S1.

**Figure 3:**
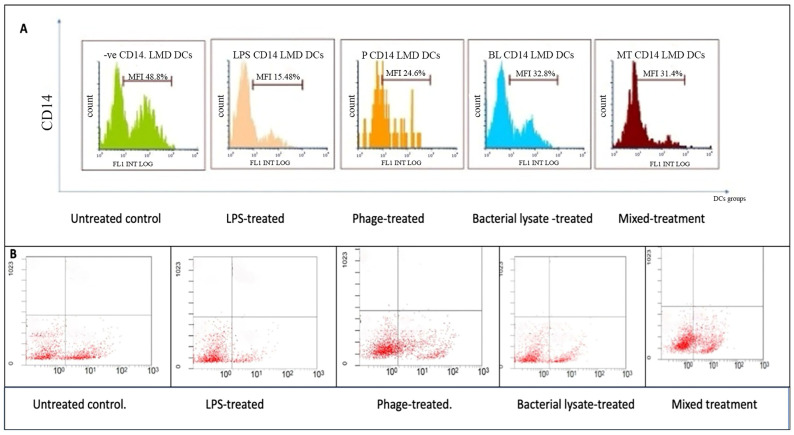
CD14 expression analysis of dendritic cells following various treatments: (**A**) representative histograms showing CD14 expression percentages in untreated (control), LPS-treated, bacterial-lysate-treated, phage-treated, and bacterial-lysate plus phage-treated DCs; (**B**) representative dot plots showing CD14-positive and CD14-negative cells among the treatment groups.

**Figure 4:**
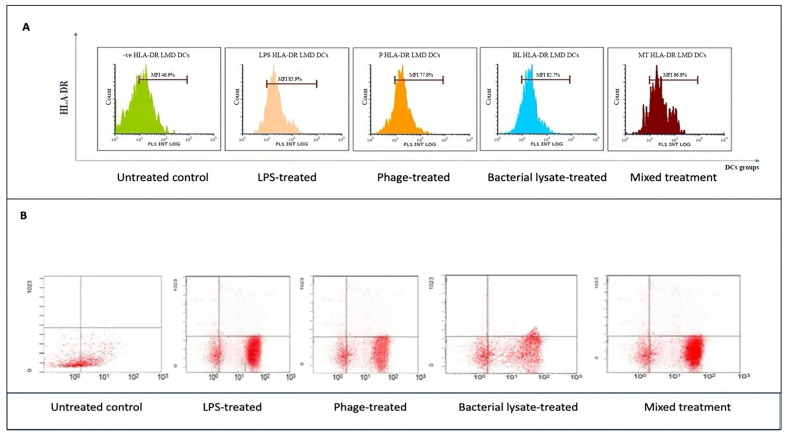
Flow cytometry analysis of HLA-DR expression in dendritic cells across different treatments: (**A**) representative histograms showing HLA-DR expression levels in untreated (control), LPS-treated, bacterial-lysate-treated, phage-treated, and bacterial-lysate plus phage-treated DCs; (**B**) representative dot plots showing the distributions of HLA-DR expression in DCs.

**Figure 5:**
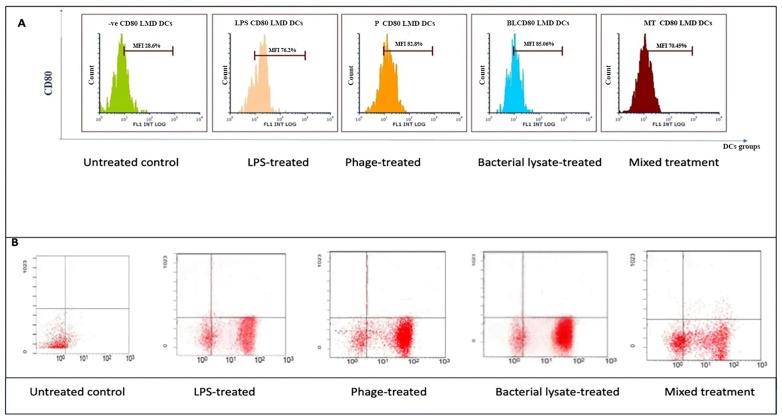
CD80 expression analysis in dendritic cells following various treatments: (**A**) representative histograms showing CD80 expression in untreated (control), LPS-treated, bacterial-lysate-treated, phage-treated, and bacterial-lysate plus phage-treated DCs; (**B**) representative dot plots depicting CD80-positive cell distributions across different treatment groups.

**Figure 6:**
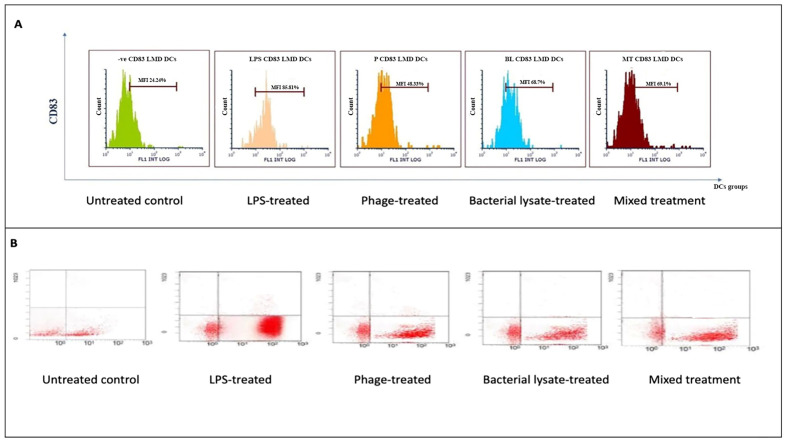
CD83 expression analysis in dendritic cells following various treatments: (**A**) representative histograms displaying CD83 expression levels in untreated (control), LPS-treated, bacterial-lysate-treated, phage-treated, and bacterial-lysate plus phage-treated DCs; (**B**) representative dot plots showing CD83-positive (B4) cell distributions in each treatment.

**Figure 7:**
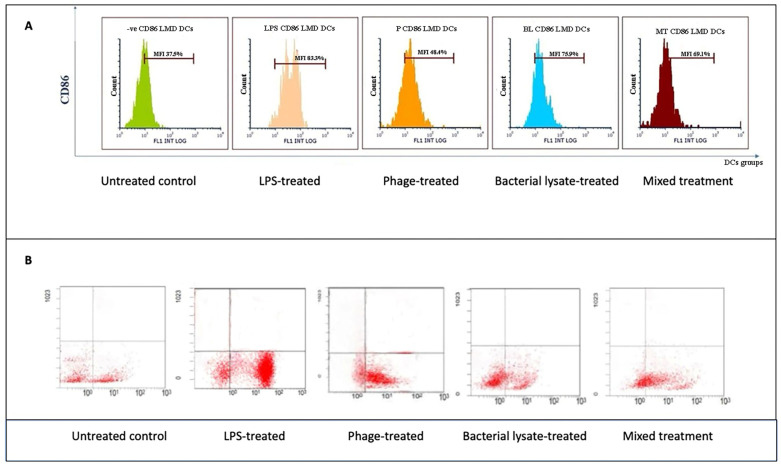
CD86 expression analysis in dendritic cells following various treatments: (**A**) representative histograms displaying CD86 expression levels in untreated (control), LPS-treated, bacterial-lysate-treated, phage-treated, and bacterial-lysate plus phage-treated DCs; (**B**) representative dot plots showing CD86-positive cells (B4) in each treatment.

**Figure 8:**
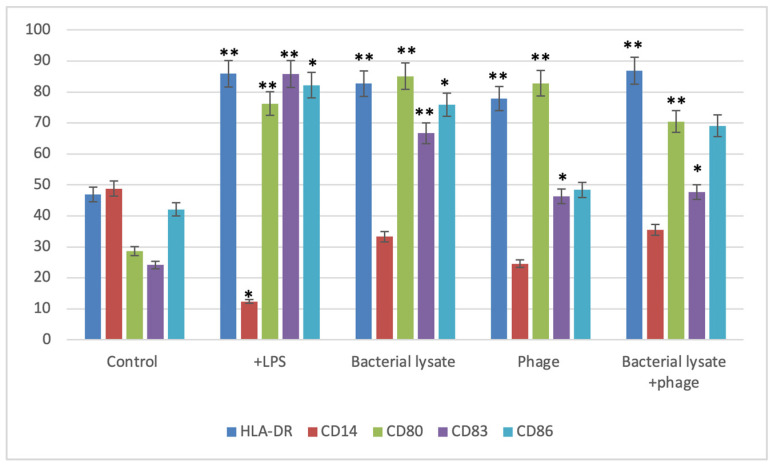
Summary of dendritic cell surface marker expression levels following various treatments. Bars represent the percentage of positive cells expressing HLA-DR, CD14, CD80, CD83, and CD86 in the control, LPS-stimulated, bacterial-lysate-treated, phage-treated, and bacterial-lysate plus phage-treated groups. Data are expressed as the mean ± SD from three independent donors. Statistical significance was determined using one-way ANOVA followed by Tukey’s post hoc test. * *p* < 0.05 and ** *p* < 0.01 vs. control group.

Figure 3, Figure 4, Figure 5, Figure 6 and Figure 7 are illustrative, not statistical. The complete statistical analysis, including *p*-values and group comparisons, is summarized in Figure 8, which consolidates all quantitative data derived from multiple experimental replicates. Flow cytometry analysis was used to evaluate CD14 expression on DCs following different treatments. The histogram overlays (Figure 3A) showed that CD14 expression in the control group was high (48.8 ± 7%). A substantial decrease was observed in the LPS-treated group (15.48 ± 12%) (*p* = 0.042), indicating efficient monocyte-to-DC differentiation. Treatment with bacterial lysate, phage, and the combination of bacterial lysate plus phage resulted in moderate reductions in CD14 expression, measuring 32.8 ± 1.8%, 24.6 ± 1.9%, and 31.4 ± 2%, respectively (statistics are shown in Figure 8).

According to the dot plot flow cytometry results (Figure 3B), CD14 expression was high (47.8%) in the control group. In the LPS-treated group, a substantial decrease (18.21%) was observed, indicating that monocytes were effectively differentiated into DCs. A combination of bacterial lysate and phage reduced CD14 expression by 30.49%, 40.65%, and 45.57%, respectively, following treatment with bacterial lysate, phage, and bacterial lysate plus phage.

HLA-DR expression, a key marker of antigen-presenting capability, was assessed by flow cytometry following various treatments. As shown in Figure 4A, HLA-DR markers in the LPS-treated DCs (85.9 ± 0.8%) were compared to those of the untreated negative control (46.9 ± 7%). Similarly, a significant increase in HLA-DR expression was observed in the bacterial-lysate-treated DCs (82.7 ± 1.2%) (*p* = 0.006). The phage-treated DCs showed a moderate upregulation (77.8 ± 2%) (*p* = 0.0078), while the combination of phage and bacterial lysate resulted in significant HLA-DR expression compared to the negative control (86.8 ± 0.7%) (*p* = 0.0089). CD14 expression remained consistently low in the treated cells, as these cells were differentiated DCs. The negative control exhibited a slightly higher CD14 expression (48.8 ± 7%). Statistics are providing in Figure 8.

As shown in Figure 4B, HLA-DR markers were lower in the DCs treated with LPSs than in the control group (85.7% compared to 45.6%). Additionally, DCs exposed to bacterial lysate increased HLA-DR expression (80.1%). Phage-treated DCs also showed upregulation (79.9%), while the combination of phage and bacterial lysate resulted in a higher HLA-DR expression (87.4%).

Following various treatments, flow cytometry analysis revealed significant modulation of CD80 expression, a key co-stimulatory molecule. The histograms (Figure 5A) show that the control group exhibited a low CD80 expression level (28.6 ± 1.5%), while LPS treatment dramatically increased CD80 expression to 76.20 ± 1.5% (*p* = 0.0069). Phage treatment also significantly elevated CD80 expression (82.8 ± 3%) (*p* = 0.037), whereas bacterial lysate and the combination of bacterial lysate plus phage led to intermediate increases of 85.06 ± 5% (*p* = 0.0062) and 70.45 ± 6% (*p* = 0.0399), respectively, as shown in Figure 8.

The dot plot analysis (Figure 5B) demonstrated that while the control group had only 20.7% of cells positive for CD80 (B4 quadrant), the LPS-treated cells showed an increased level (72.7%), reflecting gating variations. However, the bacterial lysate (76.5%), phage (84.3%), and bacterial lysate plus phage (61.8%) treatments displayed increased CD80-positive populations compared to the untreated controls.

CD83 expression, a dendritic cell (DC) maturation marker, was evaluated following various treatments. The histograms (Figure 6A) show that the control group exhibited low CD83 expression (24.24 ± 3%). Treatment with LPSs markedly increased CD83 expression to 85.81 ± 0.4% (*p* = 0.00067). Bacterial lysate treatment significantly elevated CD83 levels to 68.7 ± 0.2%, whereas the phage and bacterial lysate plus phage treatments led to increases of 48.33 ± 1.09% (*p* = 0.046) and 69.1 ± 4% (*p* = 0.038), respectively (Figure 8).

The dot plot analysis (Figure 6B) demonstrated a slight increase in the CD83-positive cell population (B4 quadrant) following treatment. The negative control group exhibited 28.6% CD83-positive cells, while the LPS, bacterial lysate, phage, and bacterial lysate plus phage treatments showed 79.2%, 75.06%, 61.8%, and 69.45% positivity, respectively.

For CD86 (Figure 7B), the cells did not received special treatment. CD86 expression was present in about 37.5 ± 5% of cells. At the same time, LPSs are known to strongly activate immune cells. Moreover, as expected, when cells are treated with LPS, the graph shifts significantly to the right; 83.3 ±18% (*p* = 0.047) of these cells now express CD86, indicating a strong activation. Bacterial lysate causes a good activation level, with 75.9 ± 2% (*p* = 0.046) of cells expressing CD86. It is not as strong as pure LPS, but there was still a clear increase compared to the untreated cells. The phage treatment did not cause a notable increase in CD86 expression, with 48.4 ± 5% of cells positive for the marker. This suggests that these phages can also stimulate immune cells. The mixed treatment showed that 69.1 ± 4% of cells expressed CD86. This is higher than the untreated control, but of interest, it is less than what we saw with the bacterial lysate or phage treatment alone in this particular representation (Figure 7A). In the dot plots, each dot represents a single plate well, and the results seem different due to individual variations. The negative control group expressed only 42.6% CD86. On the other hand, the treatment groups showed high expression of CD86 (80.3%, 57.9%, 69.9%, and 64.7% with LPS, bacterial lysate, phage, and mixed treatment, respectively) (Figure 8).

Figure 8 is a summary of the expression of CD83, CD80, and CD86 co-stimulatory molecules and CD14 with each treatment (LPS-treated, bacterial-lysate-treated, phage-treated, and mixed treatment) compared with the negative controls of these surface markers.

### 3.5. Effect of E. coli Phage on Cytokine Secretion

#### 3.5.1. IL-10 Cytokine Secretion

**Figure 9:**
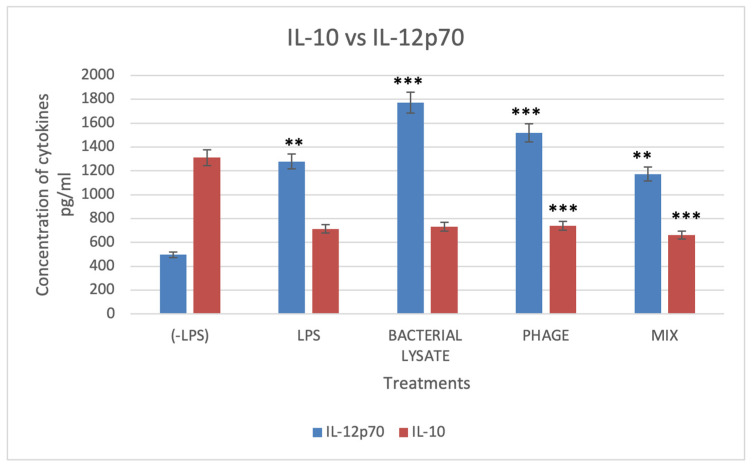
Levels of IL-10 and IL-12p70 cytokines. DCs’ culture supernatant was assessed for cytokine production upon stimulation with different treatments, determined by the ELISA technique. Data were collected from three independent experiments. * Refers to a significant difference compared with the control, where ** (*p* < 0.01), and *** *p* < 0.001.

The cytokine IL-10 secretion was measured in DCs stimulated with LPS, phage, bacterial lysate, or a combination of phage and bacterial lysate (Figure 9). Interestingly, DCs treated with bacterial lysate and phage secreted 663.6 ± 51.7 pg/mL of IL-10. In contrast, DCs treated with bacterial lysate alone secreted 733 ± 32.9 pg/mL, while phage-treated DCs secreted 740 ± 25.11 pg/mL. The control group exhibited a significantly higher IL-10 secretion level of 1311.5 ± 69.4 pg/mL. The DCs treated with bacterial lysate, phage, or their combination therapy showed a significant (*p* < 0.001) reduction in IL-10 secretion (Figure 9).

#### 3.5.2. IL-12p70 Cytokine Secretion

The pro-inflammatory cytokine IL-12p70 is secreted as dendritic cells (DCs) mature, a process induced by lipopolysaccharides (LPSs), resulting in enhanced immunostimulatory abilities. Compared to the negative control cells, which secreted 496.6 ± 136 pg/mL of IL-12p70, LPS-treated DCs secreted significantly higher levels (1278.8 ± 110 pg/mL).

In contrast, compared with the control group, DCs treated with phage, bacterial lysate, and a combination of phage and bacterial lysate secreted a significant amount of IL-12p70 (1519.8 ± 156.6, 1771 ± 89, and 1173 ± 194 pg/mL, respectively) (*p* < 0.001) (Figure 9). These cytokine profiles correlated with surface marker changes, indicating enhanced DC maturation following treatment with phage, bacterial lysate, or both. This maturation was further characterized by a shift toward a proinflammatory phenotype, as reflected by reduced IL-10 and elevated IL-12 secretion.

## 4. Discussion

Bacteriophages have numerous applications in various fields, including healthcare, veterinary science, medicine, and industry [21]. One of the most essential uses of phage is in therapy. Phage therapy is being explored as an alternative to antibiotics. Due to the growing threat of antimicrobial resistance, there is a rising demand for novel and effective treatments for antibiotic-resistant bacterial infections [22]. Antimicrobial resistance poses a significant threat to human healthcare, animal industries, food manufacturing, and agricultural sectors [23]. Clinical and safety trials have shown that phage therapy is highly effective in treating potentially fatal multidrug-resistant bacterial infections, supporting renewed interest in this area [24]. Phage therapy has excellent potential, but it also has inherent limitations. The immunogenicity of phages can affect pharmacokinetics, biodistribution, and overall therapeutic outcomes [3]. Unlike conventional phage studies, which are limited to bacterial host range or genomic profiling, our investigation explores the immunomodulatory capacity of a novel phage on primary human immune cells. This dual characterization offers a broader perspective on how phage therapy may influence not only pathogen clearance but also immune activation or tolerance, which is critical for its safe clinical translation. Thus, exploring other related areas, particularly DC differentiation, is essential. Our study aimed to elucidate the potential effects of bacteriophages on dendritic cell (DC) differentiation and provide insights into the connection between phages and DC immunity, as this could offer a valuable strategy for treating bacterial infections using bacteriophages. GADS24 is a newly isolated lytic *E. coli* phage, and our group submitted its complete genome sequence to GenBank under accession number OQ703618. BLASTn analysis showed no high-identity matches to previously reported phages across the whole genome, confirming that GADS24 represents a novel isolate. This study is the first to characterize its biological activity, particularly its immunomodulatory effects on dendritic cells.

The complete genome of GADS24 is composed of 168,982 base pairs with a GC content of 44.9%. A total of 268 open reading frames (ORFs) were predicted, of which 137 were assigned putative functions. These include genes encoding structural proteins (e.g., tail fiber and capsid), lysis-related proteins (e.g., endolysin and holin), and DNA replication elements (e.g., DNA polymerase and helicase). No genes associated with lysogeny, toxin production, or antibiotic resistance were identified, supporting its suitability for therapeutic use. The genomic profile of GADS24 aligns with that of strictly lytic myoviruses, with no evidence of lysogenic or virulence-associated genes. This supports its classification as a virulent phage, which is consistent with the clear plaque morphology and vigorous lytic activity observed in vitro. Its genome encodes hallmark proteins required for host recognition, penetration, DNA replication, and lysis, suggesting a well-adapted lytic lifecycle. The absence of undesirable genes enhances its potential for use in phage therapy or immunomodulation studies, particularly given the safety concerns surrounding temperate phages in clinical applications.

The activity of GADS24 was tested on different available strains of *E. coli*, including 9, 16, 20, 21, 30, 33, 40, 53, 401, and 882999, in which it showed its lytic action on strains 16, 21, 33, 40, and 53. These data confirm the specificity of phages to special hosts [1,2].

In this study, PEG 8000 precipitation was utilized due to its simplicity and effectiveness in concentrating bacteriophages from large sample volumes. Other purification methods, such as CsCl density gradients, can severely affect the number and activity of the phage [15,23].

LPSs are an endotoxin found in bacterial lysate. In this study, LPS levels were measured and decreased, as reported in [16,24], to 0.04 EU/mL in the phage treatment to evaluate the impact of the bacteriophage. This is a crucial step to ensure that the immunomodulatory effects observed in the “phage-treated” group are primarily due to the phage particles themselves and not due to contaminating LPSs from the phage purification process. On the other hand, when Gram-negative bacteria like *E. coli* are lysed, LPSs (an integral part of their outer membrane) are naturally released into the surrounding environment along with other bacterial components (proteins, DNA, cell wall fragments, etc.). The “bacterial lysate” group is likely intended to mimic the cocktail of bacterial components that immune cells would encounter when phages lyse bacteria in vivo or at a site of infection. A control-positive treatment of LPSs and bacterial lysate (0.37 EU/mL) was used for a complete assessment.

Dendritic cells play a crucial role in antigen presentation, initiating primary immune responses, stimulating MHC-restricted T cells, and generating T-cell-dependent antibodies [25]. In this study, GM-CSF and IL-4 were used to isolate and produce DCs from human peripheral blood. GM-CSF efficiently supports DC development in vitro, particularly from human monocytes [26,27]. High IL-4 concentrations promote the development of precursor cells into mature DCs [26,28]. A positive control, LPS, was employed to stimulate DCs and aid in maturation. LPSs are a well-known pathogen-associated molecular pattern (PAMP) molecule recognized by Toll-like receptor (TLR) 4 in monocytes and DCs. It triggers the release of pro-inflammatory cytokines and other soluble mediators, supporting our approach [29]. A recent study has demonstrated that bacteriophages, while obligate bacterial parasites, can significantly influence the immune responses in eukaryotic hosts by interacting with host DNA-sensing pathways. Specifically, bacteriophage DNA is recognized by TLR9, which activates pro-inflammatory responses while also triggering the cGAS-STING pathway, albeit with limited effectiveness [30].

The activation of TLR9 by bacteriophage DNA can induce an indirect immune response, which, while potentially pro-inflammatory, may also enhance the immune system’s ability to combat infections. DCs, as key mediators of immune responses, play a crucial role in this process. By recognizing phage DNA via TLR9, DCs can become activated and promote antigen presentation, thereby bridging innate and adaptive immunity. This indirect immune stimulation, facilitated by DCs, may help overcome some of the challenges associated with phage therapy, such as bacterial resistance and immune evasion. Thus, while TLR9 activation by phage DNA poses potential risks of inflammation, it also contributes to the therapeutic efficacy of phages by enhancing immune responses. This dual role highlights the need for further research to optimize phage therapy and balance its immune-modulating effects [6].

Flow cytometry was employed to examine the phenotypic characteristics of each treatment group. Cytometry analysis is essential for accurately identifying target cell fractions, especially when cell types such as DCs, macrophages, and monocytes share similar surface markers [31,32]. Consequently, we used HLA-DR, CD80, CD86, CD83, and CD14 as markers for DC maturation and identification. High expression of antigen-presenting markers, such as HLA-DR, indicates mature DCs. DCs were identified by the absence of the monocyte marker CD14 expression and the high expression of co-stimulatory molecules CD83, CD80, and CD86 [33]. Co-stimulatory molecules expressed by mature DCs and other APCs, including CD80 and CD86, bind to CD28 on resting T cells, facilitating the release of IL-6 and IFN-γ, as well as B-cell activation. In our study, the expression of the HLA-DR marker in the LPS-treated DCs was significantly higher compared to the negative control group, consistent with a previous report [34]. Compared to the negative control, the LPS-treated DCs expressed higher CD80, CD83, and CD86 markers, aligning with prior findings [35]. In the negative control group, CD14 expression reached 48.8%, which is considered a high level. CD14 is a monocyte marker that typically decreases as monocytes differentiate into dendritic cells (DCs), as observed in this study [36].

Rigorous statistical analyses were performed to accurately interpret the immunophenotypic changes observed in the dendritic cells following various treatments. Given the multiple treatment groups compared against each other (control, LPS, bacterial lysate, phage, and bacterial lysate plus phage), a one-way ANOVA was initially conducted to detect overall differences among groups. A post hoc Tukey honestly significant difference (HSD) test was employed to identify specific group differences while controlling for multiple comparisons. Tukey HSD provided adjusted *p*-values, ensuring a robust statistical approach that minimized the risk of Type I errors and preserved the overall confidence level. This method accurately identified which treatments significantly altered marker expression levels, supporting the biological interpretations drawn from the flow cytometry results. The statistical rigor provided by the Tukey HSD test reinforces the reliability of the findings and enhances the credibility of the observed immunomodulatory effects of the phage, bacterial lysate, and their combination. The phage-treated DC group showed significant upregulation of HLA-DR, CD80, and CD83 markers (77.8 ± 2, 82.8 ± 3, and 46 ± 3) compared to the negative control group, contrasting with results reported previously [37]. However, this result may be consistent with the research demonstrating that bacteriophages stimulate TLR9, promoting IFN-γ production. In some studies involving germ-free mice treated with purified bacteriophages (3 × 10^7^ PFU/mL), CD8+ T cells and IFN-γ levels increased considerably. CD4+ T cells were induced to produce IFN-γ by DCs activated by bacteriophage DNA. Podlacha et al. [38] suggested that the purity of preparation, particularly regarding LPS contamination, may affect the extent of bacteriophage-induced immune responses. This could explain the higher expression of these markers. Recent studies have shown that phages from individuals with type 2 diabetes (T2D) have an increased capacity to activate DCs and stimulate an IFN-γ response, suggesting that the gut virome may influence immune activity. This aligns with our findings, in which phage-treated DCs upregulated activation markers, indicating direct interaction with the immune system. These observations emphasize the complexity of phage therapy, as its pro- or anti-inflammatory effects could significantly impact treatment outcomes and immune responses [39].

DCs treated with bacterial lysate expressed HLA-DR, CD80, CD83, and CD86 at the highest levels. The presence of LPSs may be connected to the observed increase in these markers. According to Taddio et al. [40], DCs treated with phages and bacterial lysate displayed intermediate marker expression levels compared to those treated with bacterial lysate or phages alone. Phage presence impacts infected bacteria’s gene expression and metabolic pathways, potentially producing anti-inflammatory compounds, such as enzymes that degrade inflammatory mediators or block LPS signaling. This finding is supported by Zhang et al. [41], who demonstrated that *Staphylococcus aureus* bacteriophages can suppress LPS-induced inflammation in bovine mammary epithelial cells by modulating inflammatory pathways. Specifically, they showed that phage treatment reduced the expression of pro-inflammatory cytokines and inhibited NF-κB signaling, a key pathway in LPS-induced inflammation. The ability of phages to temper the immune response while still promoting bacterial clearance is particularly relevant in the context of phage therapy. Excessive inflammation, often driven by bacterial components like LPS, can lead to tissue damage and poor clinical outcomes. By modulating the immune response, phages may help achieve a balance between adequate infection clearance and controlled inflammation, enhancing the therapeutic potential of phage therapy.

DC regulation and immune response depend on IL-10 and IL-12. Activated DCs are the primary source of the pro-inflammatory cytokine IL-12, which promotes the growth of T helper 1 (Th1) cells and improves their effector function. In response to IL-12 activation, Th1 cells release more IFN-γ, enhancing the cellular immune response against intracellular infections. In contrast, IL-10, an anti-inflammatory cytokine produced by DCs and other immune cells, regulates the negative immune response of DCs. It reduces the production of pro-inflammatory cytokines, including IL-12, and the activation and maturation of DCs [41,42].

To determine whether bacteriophages may activate immature DCs, we measured IL-12 and IL-10 production in mDCs exposed to phage. Following phage and bacterial lysate treatment, the cytokine analysis of DCs’ supernatant revealed a significant increase in IL-12 production and a notable decrease in IL-10 secretion. Conversely, the bacterial lysate contained the highest concentration of IL-12 (1771 ± 89.8) [43]. Evidence suggests that the presence of LPSs in the bacterial lysate contributed to this elevated production of IL-12, consistent with findings by Van Belleghem et al. and Hamza et al. [6,44]. Additionally, the supernatant from phage-treated DCs significantly stimulated DCs’ maturation, in agreement with a previous study [45]. Unlike many previously studied *Escherichia coli* bacteriophages, such as T4, phiEco32, and vB_EcoM-VR20, which have been extensively characterized for their lytic capabilities and genomic features [46,47,48], GADS24 distinguishes itself by being evaluated for its immunomodulatory effects on human dendritic cells. While T4 and vB_EcoM-VR20 are both members of the *Myoviridae* family and share structural similarities with GADS24, they have not been investigated for their interaction with host immune cells.

In contrast, our study provides the first evidence that GADS24 can influence DC maturation by upregulating key surface markers, such as CD80, CD83, and CD86, as well as modulating pro-inflammatory cytokines, including IL-12 and TNF-α. This adds a novel dimension to phage research, as most existing studies focus solely on bacterial killing without considering host immune interactions. Furthermore, while earlier phages, such as phiEco32, were studied in the context of laboratory strains like *E. coli* K-12 [47], GADS24 was tested against a panel of clinically relevant, multidrug-resistant *E. coli* isolates. Collectively, these features position GADS24 as a promising dual-function agent, effective in bacterial clearance and capable of influencing immune pathways, thereby expanding our understanding of phage–host dynamics and informing future immunotherapeutic strategies.

The combined effect observed in the phage- and lysate-treated groups likely results from the interaction of multiple immunostimulatory components. Bacteriophages, though not inherently pathogenic to mammalian cells, can interact with pattern-recognition receptors (PRRs) such as TLR9 (via unmethylated CpG DNA motifs) or TLR3/7 (via RNA fragments), thereby triggering intracellular signaling cascades in dendritic cells [49,50]. Concurrently, bacterial lysates contain a diverse range of PAMPs, such as LPSs, peptidoglycan, and bacterial DNA, which are potent activators of innate immunity through TLR2, TLR4, and NOD-like receptors. The simultaneous presence of both phage particles and bacterial debris may, therefore, create a more robust or synergistic activation environment, leading to enhanced expression of maturation markers (e.g., CD83 and CD86) and increased cytokine production. This interplay could mimic natural conditions where phages act within infected tissues rich in bacterial remnants, thereby influencing immune cell behavior more dynamically than single-agent exposures.

The dual antibacterial and immunomodulatory properties demonstrated by GADS24 open promising avenues for its therapeutic application. Phage therapy combined with immunomodulation could offer a two-pronged strategy, as follows: directly eliminating MDR pathogens while enhancing the host’s immune defenses. Such an approach may reduce antibiotic dependency, particularly in clinical settings burdened by recurrent bacterial infections. Together, these findings support the dual role of GADS24 as both an antimicrobial and an immune modulator, with potential applications in enhancing host immunity against MDR bacterial infections. The findings from this in vitro study provide foundational knowledge for subsequent in vivo and potential clinical applications of phage therapy. Specifically, our observations of DC maturation modulation and changes in cytokine secretion by the bacteriophage GADS24 suggest that phage therapy may not only act through direct antibacterial activity but also significantly impact host immunity. Future studies involving animal models should investigate whether the observed dendritic cell responses correlate with enhanced clearance of multidrug-resistant bacterial infections in vivo.

Additionally, clinical trials are necessary to evaluate the therapeutic efficacy, optimal dosage, administration routes, safety profiles, and immunological outcomes associated with the use of GADS24 and similar phages as adjunct therapies against antibiotic-resistant pathogens. While the dual antibacterial and immunomodulatory potential of phages such as GADS24 holds great promise, several practical challenges remain before clinical translation can be realized. One primary concern is immunogenicity, as repeated or systemic phage administration may elicit neutralizing antibodies or unintended inflammatory responses in certain patients. Additionally, the route of delivery (e.g., intravenous, intranasal, or mucosal) can significantly influence both biodistribution and immune interaction, yet standardized protocols are lacking. Regulatory frameworks for phage therapy are also still evolving in many countries, which may delay approval and implementation. Finally, individual variability in immune system reactivity to phage components further complicates the predictability of therapeutic outcomes. Future studies addressing these translational barriers will be crucial for advancing phage-based immunotherapies toward clinical application.

Future research should focus on in vivo validating GADS24’s efficacy and safety using animal models and ex vivo humanized tissue systems. Further investigations into the phage’s interaction with innate immune cells, pharmacokinetics, and long-term immunological impacts will be essential for advancing GADS24 toward clinical application. A limitation of this study was the absence of heat-inactivated phage controls. Future studies should incorporate heat-inactivated bacteriophages to differentiate the immunomodulatory effects attributed specifically to active bacteriophage infectivity from non-specific structural interactions.

## 5. Conclusions

The impact of different treatments on the expression of CD80, CD83, and CD86, key markers of DC maturation, was evaluated. DCs treated with the bacterial lysate showed a considerable increase in expression of all markers, including HLA-DR, CD80, CD83, and CD86, compared with untreated DCs. Phage treatment modified the effects of bacterial lysate, with the combined treatment producing different outcomes compared with bacterial lysate alone. The results show a significant increase in IL-12 release in the DCs treated with bacterial lysate, highlighting the possible use of the bacteriophage GADS24 as an antibacterial agent against *E. coli* [51].

The available data on these interactions remain patchy, incomplete, and restricted to a small number of phages, cell types, and disease models. Unlike cells or animal models, there is also a lack of definitive data showing that phages have a direct influence on human health or immunity. Furthermore, many specific mechanisms underlying the mammalian immune response to phages are still unknown. It is still unknown how phages are taken up by cells, the mechanisms required to trigger host immunity, and whether they are initiated by phage uptake or surface interaction. Clinical studies are crucial to finding answers to these questions and may also facilitate the development of novel, phage-based therapies. While there is still much to learn about phage–mammal interactions, it is evident that this is an exciting and promising area of research. Further research is needed to analyze the effects of phages on immune cells and cell lines through additional experiments. In vivo testing is highly recommended to assess the effectiveness of phage therapy and identify its possible applications. Such tests will provide critical insights into optimizing phage therapy for various therapeutic scenarios, ultimately facilitating its broader application and development.

## Figures and Tables

**Table 1:** Host range determination for the phage GADS24.

Bacterial Strain *	Susceptibility to Isolated Phage
*E. coli* strain 53	+
*E. coli* strain 33	+
*E. coli* strain 16	+
*E. coli* strain 30	−
*E. coli* strain 21	+
*E. coli* strain 9	−
*E. coli* strain 401	−
*E. coli* strain 882999	−
*E. coli* strain 20	−
*E. coli* strain 40	+

* All *E. coli* strains listed in Table 1 were previously isolated and identified by the Special Infectious Disease Unit at the King Fahd Medical Research Center, King Abdulaziz University. All strains were confirmed by biochemical profiling and 16S rRNA sequencing. No duplicate isolates were included. All host range tests were performed in triplicate. The results represent consistently lytic activity observed across independent replicates.

## Data Availability

The original contributions presented in this study are included in the article; further inquiries can be directed to the corresponding author.

## Supplementary Materials

The following supporting information can be downloaded at: https://www.mdpi.com/article/10.3390/biomedicines13071519/s1. Figure S1. Representative gating strategy used in flow cytometric analysis of dendritic cell marker expression: (**A**) left plot—dot plot of unstained dendritic control cells, utilized to establish baseline fluorescence and gating thresholds; (**B**) right plot—dot plot showing dendritic cells stained for HLA-DR, clearly demonstrating the gating approach used to differentiate positive from negative cell populations. This gating strategy was consistently applied across all experiments to analyze additional dendritic cell markers (CD80, CD83, and CD86).

## Abbreviations

The following abbreviations are used in this manuscript:

AMR	Antimicrobial Resistance
ANOVA	Analysis of Variance
CD	Cluster of Differentiation (e.g., CD80, CD83, CD86, and CD14)
cGAS-STING	Cyclic GMP-AMP Synthase—Stimulator of Interferon Genes
DCs	Dendritic Cells
*E. coli*	*Escherichia coli*
ELISA	Enzyme-Linked Immunosorbent Assay
FSC/SSC	Forward Scatter/Side Scatter
GM-CSF	Granulocyte-Macrophage Colony-Stimulating Factor
HLA-DR	Human Leukocyte Antigen—DR Isotype
HSD	Honestly Significant Difference (Tukey’s HSD test)
IFN-γ	Interferon-Gamma
imDCs	Immature Dendritic Cells
LAL	Limulus Amebocyte Lysate
LB broth/agar	Luria Bertani Broth/Agar
LPSs	Lipopolysaccharides
MDR	Multidrug-Resistant
MHC	Major Histocompatibility Complex
OD	Optical Density
PBMCs	Peripheral Blood Mononuclear Cells
PEG	Polyethylene Glycol
PFU	Plaque-Forming Units
PI	Propidium Iodide
SD	Standard Deviation
SM buffer	Suspension Medium buffer
Th1	T helper 1 (cells)
TLR	Toll-Like Receptor (e.g., TLR4 and TLR9)
