# Supplementary figures and images for: Immunomodulatory Effects of Escherichia coli Phage GADS24 on Human Dendritic Cells
Source: Biomedicines. 2025 Jun 21;13(7):1519. doi: 10.3390/biomedicines13071519 (PMC12292600; doi:10.3390/biomedicines13071519)

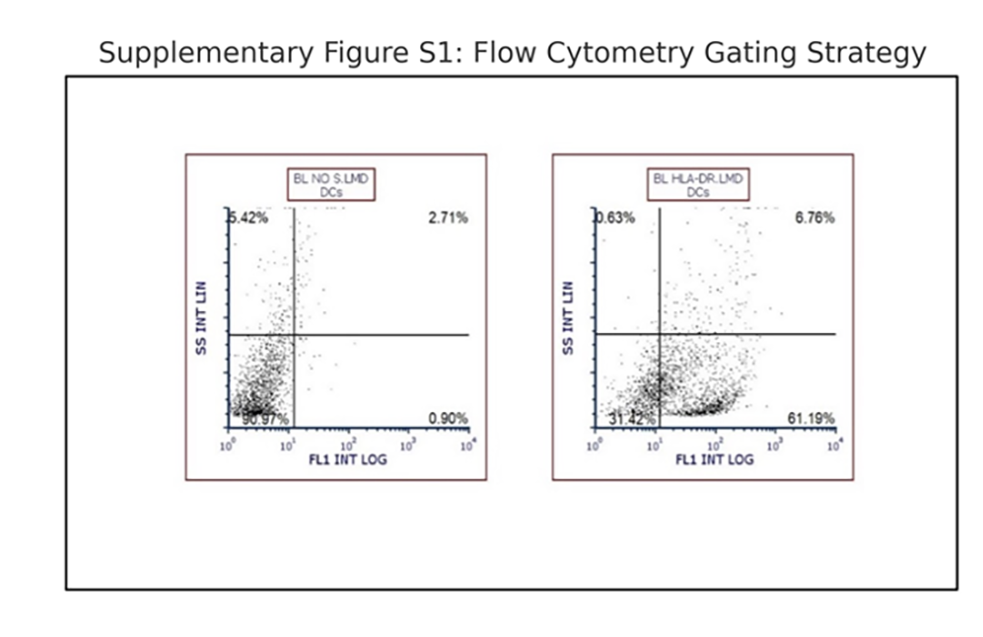

Supplement: Supplementary file 1 [file biomedicines-13-01519-s001.zip › biomedicines-3667338-supplementary.png]
